# ESR Essentials: characterisation and staging of adnexal masses with MRI and CT—practice recommendations by ESUR

**DOI:** 10.1007/s00330-024-10817-1

**Published:** 2024-06-07

**Authors:** Giacomo Avesani, Camilla Panico, Stephanie Nougaret, Ramona Woitek, Benedetta Gui, Evis Sala

**Affiliations:** 1 Department of Imaging and Radiotherapy, Fondazione Policlinico Universitario Agostino Gemelli IRCCS, Rome, Italy; 2 Department of Radiology, PINKCC Lab, IRCM INSERM, SIRIC, Montpellier, France; 3 Research Centre for Medical Image Analysis and Artificial Intelligence, Danube Private University, Krems, Austria; 4 Università Cattolica del Sacro Cuore, Rome, Italy

**Keywords:** Ovarian neoplasms, X-ray computed tomography, Magnetic resonance imaging, Patient care, Workflow

## Abstract

**Abstract:**

Ovarian masses encompass various conditions, from benign to highly malignant, and imaging plays a vital role in their diagnosis and management. Ultrasound, particularly transvaginal ultrasound, is the foremost diagnostic method for adnexal masses. Magnetic Resonance Imaging (MRI) is advised for more precise characterisation if ultrasound results are inconclusive. The ovarian-adnexal reporting and data system (O-RADS) MRI lexicon and scoring system provides a standardised method for describing, assessing, and categorising the risk of each ovarian mass. Determining a histological differential diagnosis of the mass may influence treatment decision-making and treatment planning. When ultrasound or MRI suggests the possibility of cancer, computed tomography (CT) is the preferred imaging technique for staging. It is essential to outline the extent of the malignancy, guide treatment decisions, and evaluate the feasibility of cytoreductive surgery.

This article provides a comprehensive overview of the key imaging processes in evaluating and managing ovarian masses, from initial diagnosis to initial treatment. It also includes pertinent recommendations for properly performing and interpreting various imaging modalities.

**Key Points:**

- *MRI is the modality of choice for indeterminate ovarian masses at ultrasound, and the O-RADS MRI lexicon and score enable unequivocal communication with clinicians*.
- *CT is the recommended modality for suspected ovarian masses to tailor treatment and surgery*.
- *Multidisciplinary meetings integrate information and help decide the most appropriate treatment for each patient*.

## Key recommendations

1. Ultrasound is the first-line technique for detecting and characterising adnexal masses, and MRI should be used to characterise all ultrasonographically indeterminate ones (level of evidence: high) [1]. The standardised O-RADS MRI lexicon is recommended to improve consistency and reproducibility, especially for less experienced readers; O-RADS MRI application provides a risk score of malignancy for adnexal masses (level of evidence: high), but a histological diagnosis  may be given to ensure unequivocal and univocal communication with clinicians (level of evidence: moderate).
2. CT of the thorax, abdomen, and pelvis is the recommended technique for preoperatively staging suspected ovarian cancer (level of evidence: high); a standardised lexicon and approach to reporting disease locations is recommended to ensure optimised treatment selection and surgical planning (level of evidence: moderate).
3. Imaging should be discussed in dedicated multidisciplinary meetings to consider possible options in the specific centre and offer a personalised treatment based on preoperative findings (level of evidence: high).

## Introduction

Ovarian masses are a heterogeneous group of diseases, ranging from benign to aggressively malignant.

Ultrasound (US) is the first-line imaging technique to evaluate suspected ovarian neoplasm, while magnetic resonance imaging (MRI) plays an essential role as a second-line examination to characterise adnexal masses indeterminate at US [2]. Given that imaging features of different ovarian entities may overlap, it is fundamental for the radiologist to be familiar with the main imaging characteristics of different histological subtypes and integrate them with clinical information and laboratory data, such as tumour markers, in order to narrow the differential diagnosis [3] and ideally reach a specific one.

When ovarian cancer (OC) is suspected at US or MRI, the International Federation of Obstetrics and Gynaecologists advocates the use of contrast-enhanced computed tomography for the evaluation of the extent of the disease since it provides clinically relevant information, including primary tumour extension, size and location of any peritoneal implants and lymph nodes (LNs). This information is crucial for treatment planning and to predict cytoreductive resectability.

**Fig. 1:**
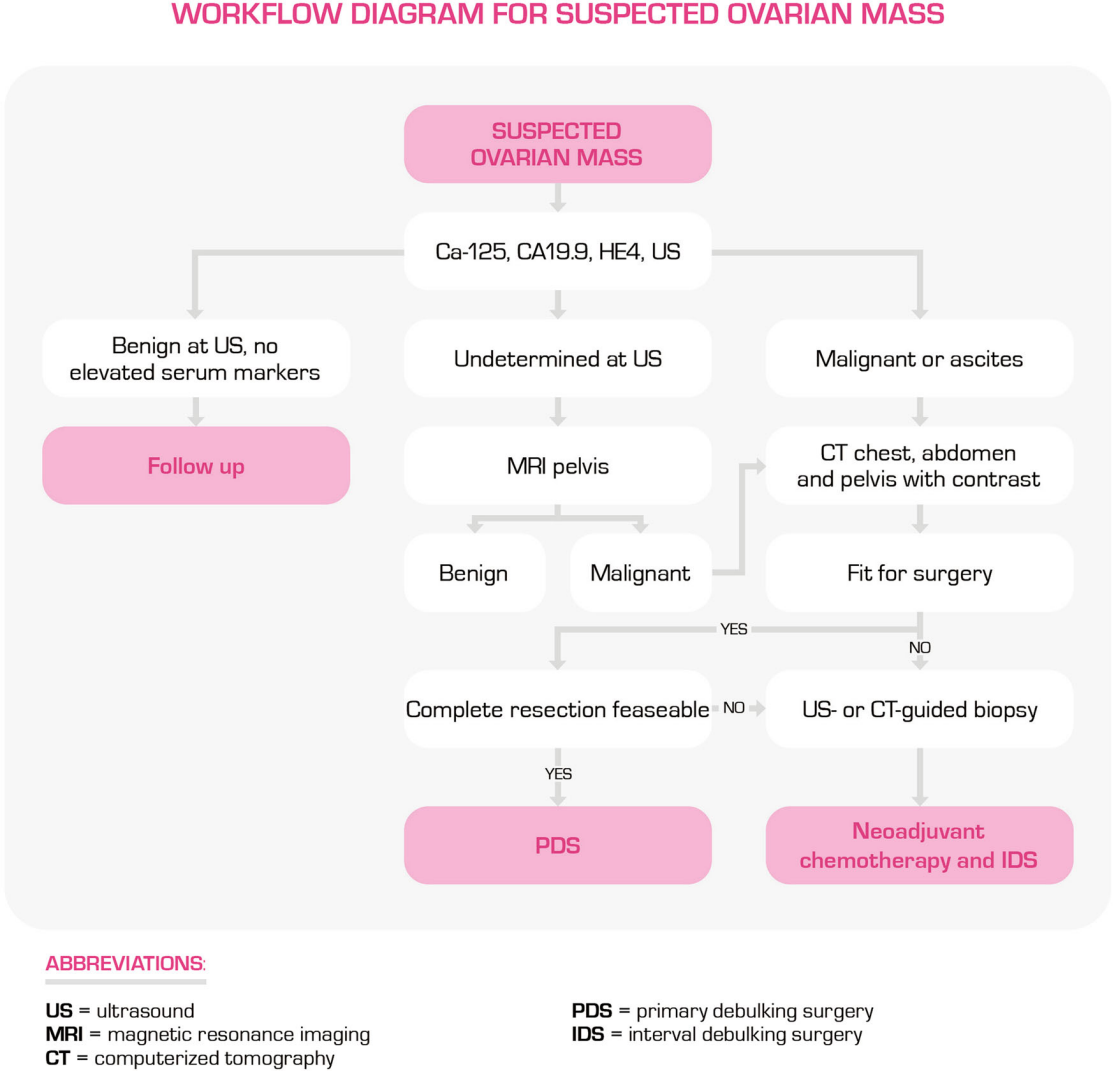
Workflow diagram for suspected ovarian lesions

Figure 1 shows the flowchart for how and when each technique is used.

## Adnexal mass characterisation

### Ultrasound

Transvaginal ultrasound (TVUS) is the first diagnostic modality for suspect adnexal masses and correctly classifies up to 80%. An in-depth explanation of the role of TVUS goes beyond the scope of this paper and detailed information can be found in publications/guidelines of the International Ovarian Tumour Analysis group and on the ovarian-adnexal reporting and data system ultrasound (O-RADS US) [4].

### Magnetic resonance imaging

#### Indication

Twenty percent of ovarian lesions are not adequately characterised in the US [5]. In such situations, MRI becomes the imaging modality of choice for further characterisation [2] due to superior contrast resolution that allows for distinguishing different tissue types.

#### Patient preparation and protocol

The MRI exam should be performed using a high-field MRI (1.5 T or 3 T), in a supine position using a surface array multichannel coil.

Fasting for 4–6 h prior to an MRI examination is no longer recommended. To reduce motion artefacts from bowel and uterine peristalsis, the administration of an antiperistaltic agent is highly suggested. Intramuscular administration has an increased length of action of approximately 30–60 min, while intravenous injection lasts 10 min. To avoid motion artefacts caused by the full bladder, it is advocated to empty the bladder 30 min before the examination.

It is crucial to optimise and standardise the MRI protocol to facilitate the characterisation of adnexal findings. A suggested protocol is provided in Table 1.

**Table 1:** Suggested MRI protocol for ovarian mass characterisations

Note: reproduced with permission from the American College of Radiology Committee on O-RADS, O-RADS Assessment Categories 2022. Available at https://www.acr.org/-/media/ACR/Files/RADS/O-RADS/O-RADS-MRI-Risk-Score_v1_2020_May-2023-_Final.pdf. Accessed on January 12, 2024

#### MRI evaluation

The O-RADS MRI score [2, 6] is the validated worldwide classification system to characterise adnexal masses based on a multivariate analysis of the most predictive features of malignancy. O-RADS MRI identifies five risk categories (Table 2) with a reported sensitivity and specificity exceeding 90% for the detection of malignancy [7].

**Table 2:** ORADS MRI risk stratification table

Note: reproduced with permission from “Sadowski EA, Thomassin-Naggara I, Rockall A, et al (2022) O-RADS MRI Risk Stratification System: Guide for assessing adnexal lesions from the ACR O-RADS Committee. Radiology 303:35–47. 10.1148/radiol.204371”

**Fig. 2:**
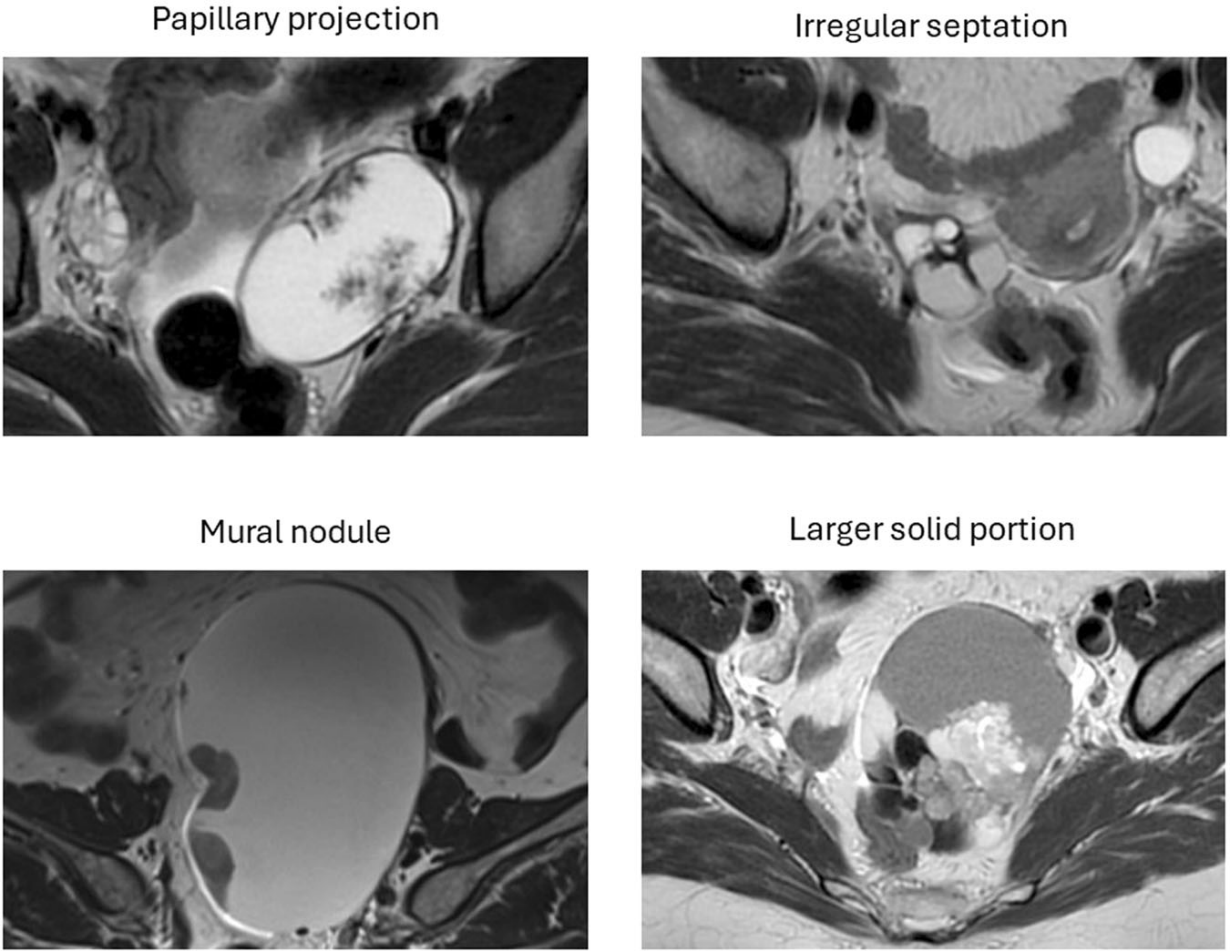
Solid tissue morphologies

A detailed overview of the O-RADS MRI lexicon has been described by Reinhold et al [8]. It is crucial to note the difference between solid components, i.e. any part of an adnexal lesion that is not simple fluid (smooth wall or septation, clot, debris, and fat), and solid tissue, which is a solid component displaying post-contrast enhancement and exhibiting the presence of at least one of the following morphologies: papillary projections, mural nodules, irregular septations or walls, and a larger solid portion (Fig. 2).

**Fig. 3:**
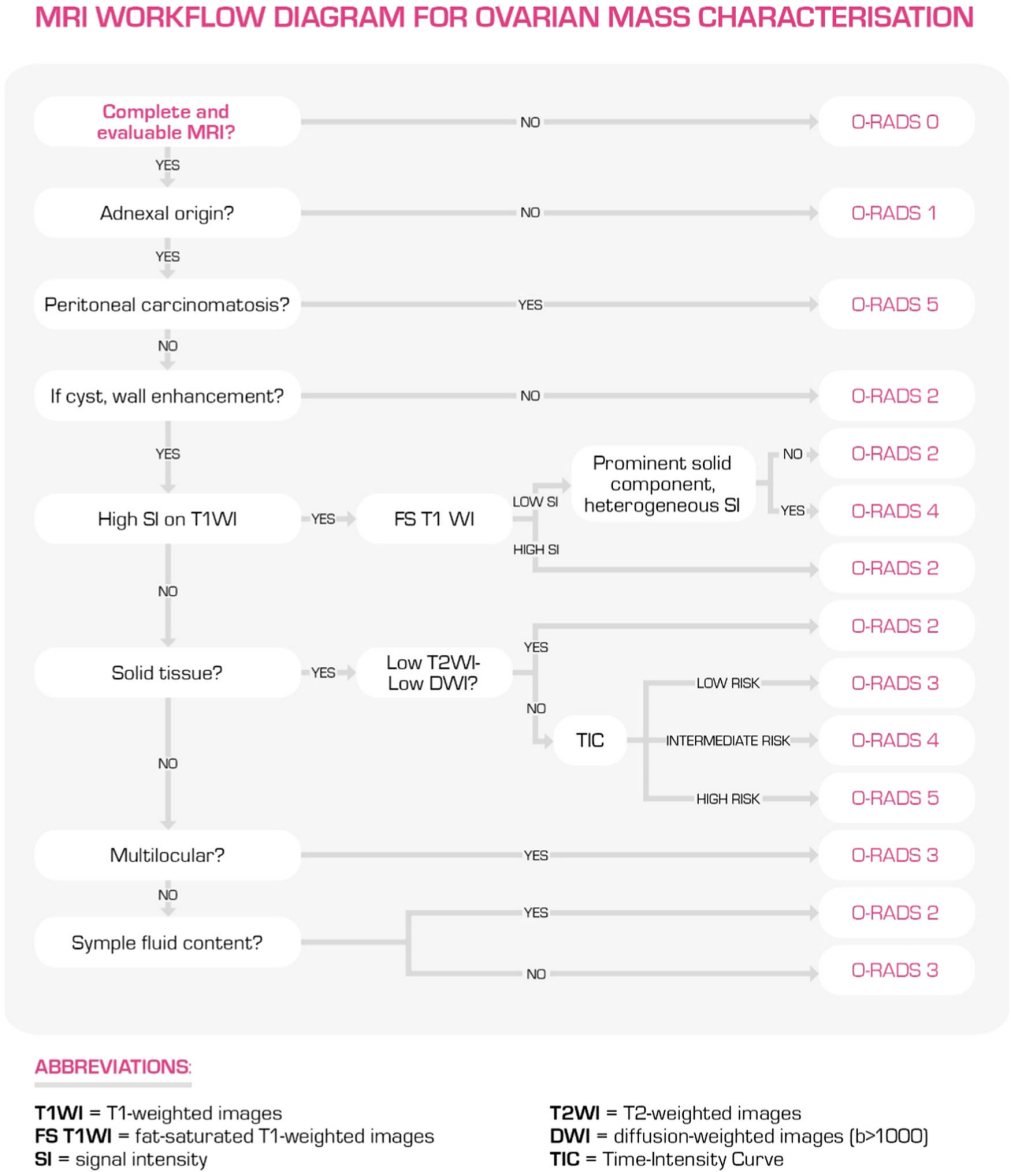
MRI Workflow diagram for ovarian mass characterisation

**Fig. 4:**
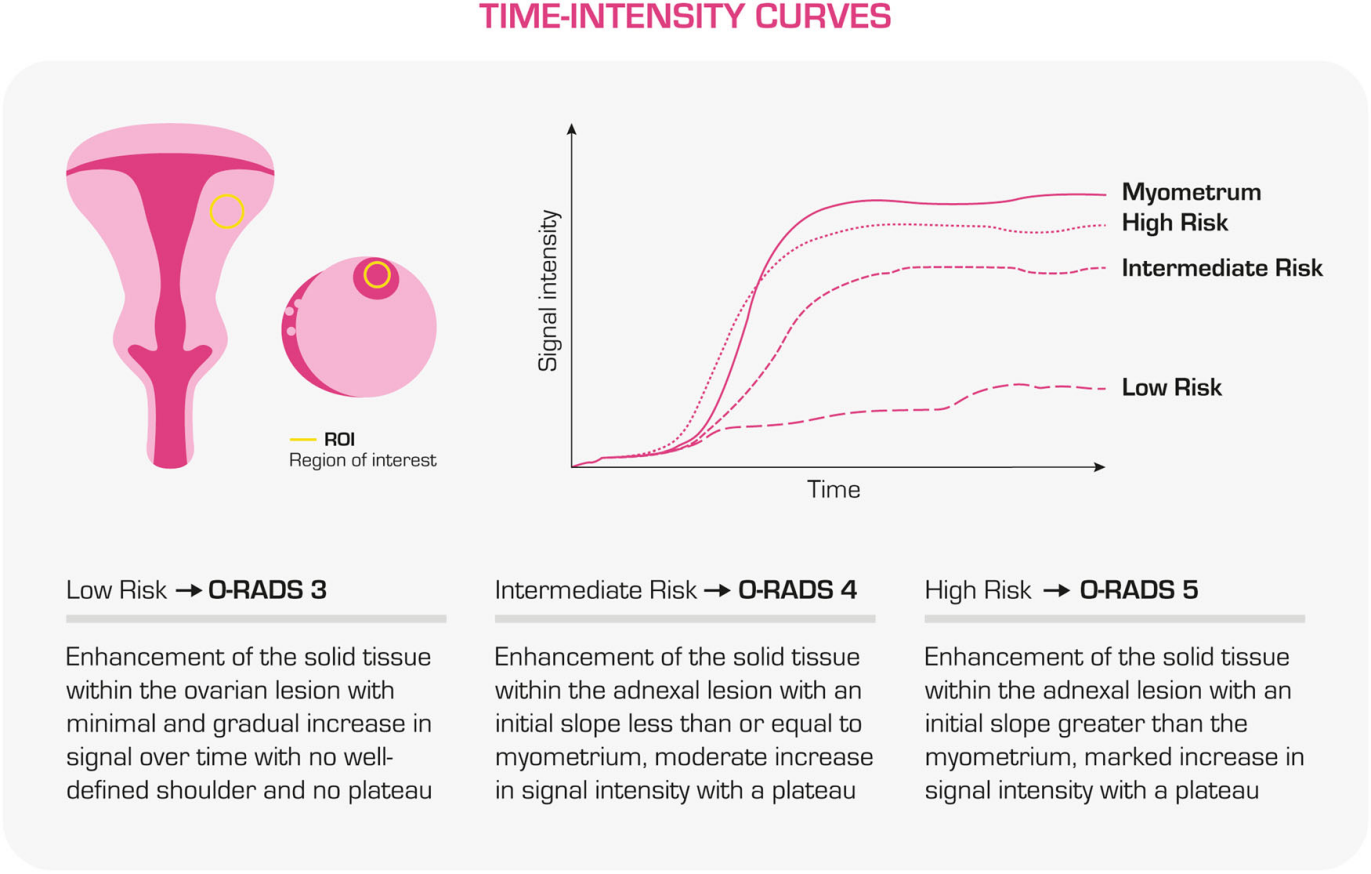
TICs and risk assessment

A diagnostic approach to MRI interpretation is proposed below (Fig. 3):

1. Identify the origin of the mass: it is fundamental to identify each ovary following the ovarian pedicle, followed by the evaluation of the lesion’s origin through the analysis of T2-weighted images (WI) in different planes since suspected indeterminate adnexal masses on the US can have different origins (uterus, gastrointestinal tract, retroperitoneum, O-RADS 1). An overview of the main MRI findings helpful in distinguishing between different origins of a pelvic mass has been reported by Forstner et al [9].
2. Verify the presence of wall enhancement in a cyst: if absent, the lesion is almost certainly benign (O-RADS 2).
3. Evaluate the signal characteristics and morphology on unenhanced T1WI and T2WI:
  T1WI hypointense and T2WI markedly hyperintense (fluid components): the presence of a unilocular cyst with a simple fluid component provides us with strong diagnostic assurance for a benign lesion (simple cyst, O-RADS 2).
  T1WI hyperintense (T1 “bright”) fluid components:
    High signal intensity (SI) on T2 and unenhanced T1WI with suppression at T1 fat-saturated images indicates the presence of macroscopic fat, while a drop of signal intensity in the opposed phase of chemical shift imaging suggests the presence of microscopic fat. Intralesional fat gives us good diagnostic confidence for a benign lesion since the vast majority of the lipid-containing mass are dermoid cysts (ORADS 2). The other less common fat-containing benign ovarian lesions are germ tumours (struma ovarii), where cystic spaces may show marked hypointensity on T2WI and intermediate signal intensity on T1WI due to thyroid colloid of the struma [10]. Immature teratomas are uncommon large malignant tumours, usually seen in adolescent women, characterised by a heterogeneous signal intensity with a prominent solid component and an infiltrative growth pattern (O-RADS 4).
    High SI on unenhanced T1WI with fat saturation reflects T1 shortening from extracellular methemoglobin, indicating the presence of blood breakdown products. If it is associated with a loss of signal intensity within the lesion on T2WI (the “shading” sign), the lesion is almost certainly an endometrioma, which is benign (O-RADS 2). On the other hand, the presence of haemorrhagic, mucinous, and proteinaceous fluid components, which show variable hyperintensity on unenhanced T1 and T2WI, indicates a low risk of malignancy (O-RADS 3).
4. Check for the presence of internal enhancement in a cyst:
  Internal enhancement corresponding to a solid tissue:
    Solid tissue with homogeneous hypointense SI on T2WI and DWI (“dark T2, dark-DWI”): This finding configures a probably benign lesion due to the presence of fibrous tissue. If the mass is predominantly solid and with homogeneous SI, the positive predictive value of malignancy is close to zero and DCE sequences are not needed (O-RADS 2; i.e. fibromas, cystadenofibromas).
    Solid tissue with intermediate SI on T2WI and restricted diffusion on DWI: contrast enhancement (CE) sequences need to be evaluated. Dynamic contrast-enhanced sequences (DCE) are recommended as they allow the building of time-intensity curves (TICs, Fig. 4) by placing one region of interest within the area of early solid tissue enhancement in the lesion and another on the outer myometrium. If DCE MRI is not feasible, non-DCE MRI can be performed as a single post-contrast T1-weighted sequence at 30 s with fat saturation and the enhancement of the solid tissue in the lesion is compared to the outer myometrium. The evaluation of the post-contrast dynamics of the solid tissue let us improve the risk assessment of the mass (O-RADS 3, 4, and 5; Fig. 4).
  Internal enhancement non-corresponding to a solid tissue: CE is probably due to thin or thick smooth septa in a bilocular or multilocular cyst or to endosalpingeal folds in a pyosalpinx (O-RADS 3).
5. Look for peritoneal carcinomatosis: if peritoneal carcinomatosis is detected, the likelihood of malignancy is high (O-RADS 5), and gynaecologic oncologist counselling is needed.

Independent from O-RADS and its risk classification, it may be important for patient management to give a histological differential diagnosis of the mass because it would give important information to plan the subsequent treatment. For example, a multilocular mucinous cystadenoma and a borderline serous ovarian tumour with papillae with an uncommon low-risk curve of enhancement might fall under the same category of O-RADS MRI 3. However, they should be treated with different priorities and possibly with different surgical approaches. To help with this, an overview of the distinguishing MRI features of different ovarian neoplasms, clinical information, and laboratory data is shown in Table 3.

**Table 3:** Imaging and laboratory characteristics of main ovarian tumours

While contrast-enhanced MRI score is preferred [2], non-contrast MRI scores have shown good accuracy in predicting the risk of malignancy [11]. This score emphasises the importance of morphological sequences and DWI for evaluating solid tissue and includes in the risk stratification ancillary features like ascites, lymphadenopathy, and peritoneal implants. Non-contrast MRI scores can be a suitable diagnostic alternative when the administration of intravenous contrast medium is not feasible, such as in pregnant women, particularly during the first trimester [12].

## Staging and surgical treatment planning

### Objectives

In OC, the established treatment is a combination of surgery for complete cytoreduction and platinum-based chemotherapy. The objective of surgery is the excision of all visible tumour localisations [13]. Incomplete resection is a strong negative prognostic factor and should be avoided. When possible, surgery is done as the first treatment (primary debulking surgery, PDS), but if the complete gross resection is considered too difficult to achieve, neoadjuvant chemotherapy followed by interval debulking surgery should be performed. A significant issue in the pre-surgical evaluation of debulking feasibility is the lack of universally accepted standardised criteria, with different individual criteria and preferences depending on the skill and experience of the surgical team. Discussion of suitability for PDS in multidisciplinary tumour boards is an essential part of treatment planning [14]. In this context, imaging to determine the extent and spread of the tumour is vital for tailoring treatment plans [15, 16].

### Imaging modalities

Currently, multidetector CT is the preferred method for staging OC due to its broad availability, relatively low cost, fast scanning time, and large coverage.

While MRI matches CT in diagnostic accuracy, it is generally considered a secondary modality for OC staging. MRI is the best modality in cases where CT is not suitable, such as for patients with allergies to contrast media, renal insufficiency/failure, pregnancy, and young patients. However, the lower availability, the longer scan time, and the higher cost limit its use. DWI is the most useful sequence in peritoneal implant detection, as they usually show restricted diffusion (high signal intensity in high *B*-value sequence and low in apparent diffusion coefficient maps), as well as an intermediate signal intensity in T2WI.

Guidelines do not suggest using [^18^F]fluorodeoxyglucose positron emission tomography for the initial diagnosis and staging of OC [17].

### Protocol

For staging advanced OC, CT scans of the chest, abdomen, and pelvis are recommended. Using intravenous iodinated contrast agents is mandatory as non-contrast CT does not provide essential information. Oral contrast is less widely accepted but recommended by the ACR appropriateness criteria [18]. A positive oral contrast agent is particularly effective in making bowel loops visible and identifying peritoneal deposits on the serosal intestinal surface and within the mesentery. However, small calcified implants, more common in low-grade serous OC, might be less visible with positive oral contrast and could be more clearly detected using a negative oral contrast like water. Additionally, examining reconstructed coronal and sagittal images alongside the standard axial images can enhance the detection of peritoneal implants.

### Main findings to report

Utilising a disease-focused approach and standardised terminology can aid radiologists in comprehensively and coherently documenting all critical disease sites [19]. The standardised lexicon proposed by the Society of Abdominal Radiology (SAR) and the European Society of Urogenital Radiology (ESUR) is recommended [20].

From a surgical standpoint, specific disease sites may pose more challenges than others to be resected, and these locations should be specifically highlighted [21].

The European Society of Gynaecologic Oncology enlists the following findings as “criteria against abdominal debulking” [22]:

- Diffuse deep infiltration of the root of small bowel mesentery.
- Diffuse carcinomatosis of the small bowel which would lead to short bowel syndrome (remaining bowel < 1.5 m) or total colectomy.
- Diffuse involvement/deep infiltration of stomach/duodenum (limited excision is possible) or pancreas head.
- Involvement of coeliac axis and its branches (coeliac nodes can be resected).

### Upper abdomen

Implants located in the fissure for ligamentum venosum (the area between the caudate and left hepatic lobes), falciform ligament, the porta hepatis, the lesser omentum (including the hepatogastric and hepatoduodenal ligaments), and those with direct liver tissue invasion often necessitate more complex liver surgery, possibly requiring the expertise of a hepatobiliary surgeon. Parenchymal invasion is suspected when unclear, nodular, or merged boundaries with the liver are noted.

In the left upper quadrant, tumours located in the splenic hilum or within the spleen may require a splenectomy. If anticipated by imaging, this procedure can be carried out along with removing the omentum.

### Bowel and mesentery

The lesion morphology and location, number of affected segments, and proximity to the bowel of mesenteric lesions are crucial for surgical decision-making. Surgeons must consider the risks of extensive bowel resection, including increased operating time and potential complications. CT scans often underestimate small bowel involvement and particular attention should be paid to this aspect.

While most mesenteric disease is resectable, implants in the central mesentery can lead to less effective debulking or higher morbidity if resection is attempted. These implants range from fat infiltration to small nodules or large, irregular masses, typically surrounding mesenteric vessels, affecting resectability. Secondary signs like bowel loop retraction, tethering, and angulation are also crucial for radiologists to identify mesenteric involvement. Discrete nodules are resectable but extend the surgery time. Diffuse infiltration and retraction, indicating central mesentery involvement, require neoadjuvant chemotherapy before surgery.

### Lymph nodes

Radiologic reports should include all abnormal LNs, although certain LN locations pose more significant surgical challenges. These include LNs in the para-aortic area above the renal vessels, near the porta hepatis, adjacent to the coeliac axis, within the mesentery, and in the retrocrural area. Cardiophrenic LNs are considered abnormal if they exceed 0.5 cm in the short axis [23] and can be resected when in the anterior position. Inguinal LNs larger than 1.5 cm are also considered abnormal but can be excised even in stage IVB cases.

### Other localisations

Other stage IV sites, like splenic parenchymal metastases, can be addressed through splenectomy. However, metastases in liver tissue, lungs, pleura, abdominal wall, or other soft tissues are usually considered not resectable except when limited or isolated.

**Fig. 5:**
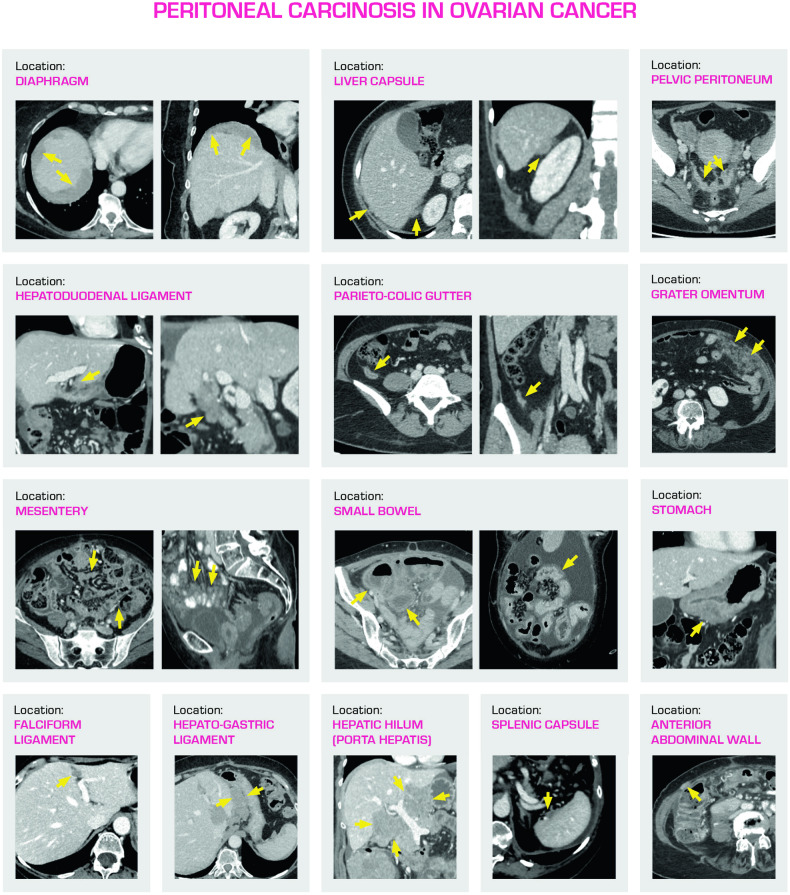
Examples of principal locations of peritoneal carcinosis to be reported

The main peritoneal locations of disease that should be reported are listed in Fig. 5, with typical examples of CT findings.

## Structured report

It is recommended to summarise MRI and CT findings in a structured report, including the size, location, imaging characteristics, and conclusion specifying any differential diagnosis, to improve consistency in reporting disease and promote communication between radiologists and clinicians [24]. An example of a structured report is in Supplementary Table 1.

## Future perspectives

In certain cases, ovarian mass characterisation, as well as subtle peritoneal implants detection, may be challenging. In recent years, many researchers have tried to implement the use of quantitative methods relying upon artificial intelligence (AI) techniques. These methodologies can analyse complex patterns related to the microstructure of tissues that may not be visible to the human eye by feature extraction, like in radiomics, eventually with automated analysis using convolutional neural networks. These techniques and the integration with other high-throughput data (genomic, proteomic, etc.) [25] may lead to more accurate diagnosis by tracking tumour evolution and treatment response. The use of AI may improve personalised therapeutic approaches, leading to more individualised treatment plans in the future [26].

## Summary statement

Imaging in the initial workup of suspected ovarian masses is fundamental.

The first-line approach should be done by TVUS, ideally performed by an expert in the field, and interpreted together with clinical and serum findings. This simple and widely available first step can solve most diagnostic problems.

In 8–30% of cases, the suspected mass is indeterminate in the US. In those cases, an MRI should be performed, which has a higher accuracy in tissue characterisation. In MRI interpretation, the O-RADS lexicon and stratification risk assessment are useful and should be applied; nonetheless, including any suspected histopathological diagnosis in the radiological report can benefit clinicians in treatment planning.

When an adnexal mass is suspected of malignancy at the US or MRI, a contrast-enhanced CT should be performed to study the thorax, abdomen, and pelvis. Also, in CT, the standard lexicon should be used, and the precise locations of the metastases should be systematically reported, possibly using a structured report. This can help with the correct interpretation and discussion of each case in a multidisciplinary tumour board, which is a fundamental step to personalising the treatment, which can differ among centres and patients.

## Patient summary

Ovarian masses are a frequent medical problem, and treatments can differ widely. Correct diagnosis and evaluation of the spread of disease are fundamental for the correct management. US is the first imaging step, followed by MRI for unclear cases. MRI differentiates tissue types and, along with the O-RADS MRI score, helps in risk assessment. When cancer is suspected, contrast-enhanced CT is used for staging and guiding treatment planning. Accurate imaging is crucial in managing OC, aiding in diagnosis, staging, and determining appropriate treatment. Management should decide on a dedicated meeting with all physicians involved in diagnosis and treatment.

## Supplementary information

Electronic Supplementary Material

## Abbreviations

OC: Ovarian cancer
O-RADS: Ovarian-adnexal reporting and data system
TVUS: Transvaginal ultrasound
