# Supplementary material for: ESR Essentials: characterisation and staging of adnexal masses with MRI and CT—practice recommendations by ESUR
Source: Eur Radiol. 2024 Jun 7;34(12):7673–89. doi: 10.1007/s00330-024-10817-1 (PMC11557651; doi:10.1007/s00330-024-10817-1)
Supplement: Supplementary file 1 — Electronic Supplementary Material [file 330_2024_10817_MOESM1_ESM.pdf]

ESR Essentials: Characterisation and staging of adnexal masses with MRI and CT– practice recommendations by ESUR

Electronic Supplementary Material

Supplementary Table 1: an example of structured report for ovarian cancer staging on CT

INDICATIONS: QUALITY: Good/limited by

COMPARISON: [None/Date]

<u>THORAX</u>

- Lung parenchyma:
  - Metastases: No/Yes
  - Other lung findings:
- Lymphadenopathy: No/Yes (location, short axis)
- Pleural effusion: No/Yes
- Pericardial effusion: No/Yes
- Other findings:

<u>ABDOMEN AND PELVIS</u>

- Adnexa:
  - Pathological lesions: No/Yes
  - dimensions (mm x mm x mm)
  - infiltration of adjacent organs: No/Yes
- Peritoneal carcinomatosis: No/Yes:
  - Diaphragm: No/Yes:
    - right: confluent (<50% / >50%) / nodular (max … mm)
    - left: confluent (<50% / >50%) / nodular (max … mm)
  - Liver capsule: No/Yes: diffuse (thickness mm) / nodular (max … mm)
  - Falciform ligament: No/Yes
  - Gastro-hepatic ligament: No/Yes
  - Gastro-duodenal ligament: No/Yes
  - Lesser sac: No/Yes
  - Hepatic hilum: No/Yes (if Yes: portal vein infiltration: No/Yes; hepatic artery infiltration: No/Yes)
  - Splenic capsule: No/Yes
  - Right parieto-colic gutter: No/Yes

    - Left parieto-colic gutter: No/Yes
    - Pelvic peritoneum: No/Yes
    - Omentum: No/Yes
    - Mesentery: No/Yes (if Yes: nodular/infiltrative (specify if there is retraction of the intestinal loops in free text)
    - Anterior abdominal wall: No/Yes
- Gastro-enteric tract: No/Yes, involvement of wall:
    - gastric: No/Yes
    - small bowel: No/Yes
    - colonic: No/Yes
    - recto-sigmoid: No/Yes
- Intestinal occlusion/sub-occlusion: No/Yes.
- Bladder involvement: No/Yes
- Ureteral involvement: No/Yes, (if Yes Dx/Sn, Hydronephrosis: No/Yes)
- Vascular involvement: No/Yes, venous/arterial
- Lymphadenopathy: No/Yes
- Ascites: No/Yes
- Abdominal organs:
    - Liver: nothing to report
    - Gallbladder and biliary tract: nothing to report
    - Pancreas: nothing to report
    - Spleen: nothing to report
    - Adrenal glands: nothing to report
    - Kidneys and urinary tract: nothing to report
- Vascular anomalies:
- Other findings:

<u>BONE</u>

- Suspicious focal lesions: No/Yes

CONCLUSIONS: Ovarian cancer FIGO stage
